# Acute injury in the peripheral nervous system triggers an alternative macrophage response

**DOI:** 10.1186/1742-2094-9-176

**Published:** 2012-07-20

**Authors:** Elke Ydens, Anje Cauwels, Bob Asselbergh, Sofie Goethals, Lieve Peeraer, Guillaume Lornet, Leonardo Almeida-Souza, Jo A Van Ginderachter, Vincent Timmerman, Sophie Janssens

**Affiliations:** 1 Peripheral Neuropathy Group, Department of Molecular Genetics, VIB and University of Antwerp, Antwerpen, Belgium; 2 Department of Biomedical Molecular Biology, Ghent University, Ghent, Belgium; 3 Department for Molecular Biomedical Research, VIB, Ghent, Belgium; 4 GROUP-ID Consortium, Laboratory for Immunoregulation and Mucosal Immunology, GhentUniversity, Ghent, Belgium; 5 Laboratory of Cellular and Molecular Immunology, Vrije Universiteit Brussel, Brussels, Belgium; 6 Myeloid Cell Immunology Lab, VIB, Brussels, Belgium

**Keywords:** Innate immune system, Negative regulation, M2, RT-qPCR, Neuroprotection, Wallerian degeneration

## Abstract

**Background:**

The activation of the immune system in neurodegeneration has detrimental as well as beneficial effects. Which aspects of this immune response aggravate the neurodegenerative breakdown and which stimulate regeneration remains an open question. To unravel the neuroprotective aspects of the immune system we focused on a model of acute peripheral nerve injury, in which the immune system was shown to be protective.

**Methods:**

To determine the type of immune response triggered after axotomy of the sciatic nerve, a model for Wallerian degeneration in the peripheral nervous system, we evaluated markers representing the two extremes of a type I and type II immune response (classical *vs.* alternative) using real-time quantitative polymerase chain reaction (RT-qPCR), western blot, and immunohistochemistry.

**Results:**

Our results showed that acute peripheral nerve injury triggers an anti-inflammatory and immunosuppressive response, rather than a pro-inflammatory response. This was reflected by the complete absence of classical macrophage markers (iNOS, IFNγ, and IL12p40), and the strong up-regulation of tissue repair markers (arginase-1, Ym1, and Trem2). The signal favoring the alternative macrophage environment was induced immediately after nerve damage and appeared to be established within the nerve, well before the infiltration of macrophages. In addition, negative regulators of the innate immune response, as well as the anti-inflammatory cytokine IL-10 were induced. The strict regulation of the immune system dampens the potential tissue damaging effects of an over-activated response.

**Conclusions:**

We here demonstrate that acute peripheral nerve injury triggers an inherent protective environment by inducing the M2 phenotype of macrophages and the expression of arginase-1. We believe that the M2 phenotype, associated with a sterile inflammatory response and tissue repair, might explain their neuroprotective capacity. As such, shifting the neurodegeneration-induced immune responses towards an M2/Th2 response could be an important therapeutic strategy.

## Background

Injury to the peripheral nervous system (PNS) induces a well-orchestrated cellular process that leads to the complete disintegration of the nerve segment distal to the lesion site, termed Wallerian degeneration (WD) [1]. As axons are disconnected from their cell bodies, they are rapidly fragmented by an intrinsic active process of self-destruction [2]. Due to the loss of axonal contact, the myelinating Schwann cells (SC) dedifferentiate into an immature phenotype, start proliferating, and help in the degeneration of myelin. Wallerian degeneration typically triggers a strong neuroinflammatory response in which the SCs are believed to play an important role. Being in close contact with the nerves, SCs are among the first to respond to nerve damage. They induce the production of pro-inflammatory cytokines such as TNF, IL-1α, and IL-1β within hours after nerve injury [3-5]. Subsequently, these cytokines induce the expression of additional immune mediators such as IL-6, GM-CSF, and IL-10 in both Schwann cells and fibroblasts [3,4,6,7]. The production of MCP-1 and MIP-1α, which reaches a maximum at 1 day after injury, promotes the recruitment of macrophages to the damaged nerves [4,8]. Moreover, mast cells accumulate in the endoneurium of injured nerves [9] and release mediators that contribute to the recruitment of macrophages and neutrophils [9,10]. Infiltration of blood-borne monocytes, which spread over the entire nerve, starts from 2 to 3 days after injury and macrophage accumulation peaks at 7 days post injury [11-14]. These infiltrated immune cells take over the cytokine production and are responsible for the rapid clearance of myelin debris. Before the infiltration of hematogenous macrophages, local macrophages proliferate and undergo morphological changes consistent with immunophenotypic signs of activation [15]. These resident macrophages, together with SCs, readily contribute to myelin phagocytosis [12,16]. Two to three weeks after injury, the inflammatory response is turned off and macrophages are rapidly eliminated [12,13,17]. Generally, WD is believed to induce a strong pro-inflammatory response, as reflected by the induction of cytokines such as TNF and IFNγ [18-20], and the reported elevation of iNOS [21]. Still, one would expect that neurodegeneration might trigger a more dampened immune response, which is typically associated with sterile inflammation. To address this question we used a model of WD (axotomy of the sciatic nerve) to analyze which type of immune response is being induced. Our results revealed that WD leads to the expression of several negative regulators of the innate immune system. In addition, a predominant M2-like macrophage response could be observed, reflecting the presence of an immunosuppressive milieu necessary to initiate wound repair and restore tissue homeostasis.

## Material and methods

### Mice work and induction of peripheral nerve injury

All animal experiments were approved by the local ethics committee (University of Antwerp), and conducted according to the guidelines of the Federation of European Laboratory Animal Science Associations (FELASA). Axotomy experiments of the *N. ischiadicus* (sciatic nerve) were conducted in 6- to 8-week-old C57BL/6 mice as previously described [22]. Briefly, mice were anesthetized with a single intraperitoneal injection of ketamine (Ketalar; Pfizer; 150 mg kg^-1^) and xylazine (Rompun; Bayer; 10 mg kg^-1^). An incision was made at the right thigh, and gluteal and hamstring muscles were carefully separated to expose the sciatic nerve. The sciatic nerve was transected and the wound was closed by sutures. The contralateral side was left untouched. For analgesia, bupronorphinum (Temgesic; Schering-Slough; 0,1 mg kg^-1^) was injected after surgery. Sham operation experiments were performed to evaluate the effect of damage around the nerve, inflicted by the operation, on the gene expression profile.

### Intravenous injection of TLR ligands

Lipopolysaccharide (LPS; TLR4-ligand) (Sigma; 10 mg kg^-1^) or triacyl lipopeptide (Pam3Cys; TLR1/2-ligand) (Sigma; 10 mg kg^-1^) were injected intravenously in 6- to 8-week-old C57BL/6 mice. PBS was injected in the control mice.

### RNA isolation and RT-qPCR

At defined time points after sciatic nerve transection, the mice were euthanized by inhalation of CO_2_. The distal part of the transected *N. ischiadicus* and the contralateral control side were removed; snap frozen and stored at −80 °C until use. The nerves were homogenized in Trizol with a Potter Elvehjem homogenisator, and small fragments were further homogenized by sonication. Total RNA was extracted using the RNeasy Lipid Tissue kit (Qiagen) according to the manufacturer’s protocol. The quality of the RNA was verified by gel electrophoresis. DNase treatment was performed with TURBO DNase (Ambion). cDNA was produced using the Superscript III first strand synthesis system for RT-PCR (Invitrogen). Real-time quantitative polymerase chain (RT-qPCR) reactions were done with 10 ng cDNA in SYBR Green I mix and run on an ABI Prism 7900 HT Sequence Detection System (Applied Biosystems). All PCR reactions were performed in triplicate. Primers were designed making use of Primerbank (http://www.pga.mgh.harvard.edu/primerbank). Primer sequences are listed in Table 1. The RT-qPCR data were normalized according to the method described by Vandesompele *et al*. [23], by geometric averaging of multiple internal control genes. Processing the raw data and normalization of the relative quantities were computed with an improved version of the ΔΔ-Ct-method [24]. The mRNA expression levels are expressed relative to the basal condition (0 h time point; not operated mice or not injected mice).

**Table 1:** Primer sequences

*Negative regulators*		
A20	GAACAGCGATCAGGCCAGG	GGACAGTTGGGTGTCTCACATT
IκBα	TGAAGGACGAGGAGTACGAGC	TTCGTGGATGATTGCCAAGTG
IL-1RA	GCTCATTGCTGGGTACTTACAA	CCAGACTTGGCACAAGACAGG
MyD88s	TGAAGTCGCGCATCGGAC	CGGCGACACCTTTTCTCAAT
SIGIRR	GTGACATGGCCCCTAATTTCC	ATGCCAGACCATCTTTCAGCC
SOCS1	CTGCGGCTTCTATTGGGGAC	AAAAGGCAGTCGAAGGTCTCG
*Immune mediators*		
Cox2	TGAGCAACTATTCCAAACCAGC	GCACGTAGTCTTCGATCACTATC
MIP-1α	TTCTCTGTACCATGACACTCTGC	CGTGGAATCTTCCGGCTGTAG
IL1β	GCAACTGTTCCTGAACTCAACT	ATCTTTTGGGGTCCGTCAACT
IL6	TAGTCCTTCCTACCCCAATTTCC	TTGGTCCTTAGCCACTCCTTC
MCP-1	TTAAAAACCTGGATCGGAACCAA	GCATTAGCTTCAGATTTACGGGT
*Macrophage markers and M1/M2 markers*		
AIF1/Iba1	ATCAACAAGCAATTCCTCGATGA	CAGCATTCGCTTCAAGGACATA
Arg1	TGGCTTGCGAGACGTAGAC	GCTCAGGTGAATCGGCCTTTT
CD11b	CCATGACCTTCCAAGAGAATGC	ACCGGCTTGTGCTGTAGTC
Cdh1	GTCTACCAAAGTGACGCTGAA	GGGTACACGCTGGGAAACAT
F4/80	ATGGACAAACCAACTTTCAAGGC	GCAGACTGAGTTAGGACCACAA
FIZZ1	CCAATCCAGCTAACTATCCCTCC	ACCCAGTAGCAGTCATCCCA
IFNγ	ATGAACGCTACACACTGCATC	CCATCCTTTTGCCAGTTCCTC
IL4	GGTCTCAACCCCCAGCTAGT	GCCGATGATCTCTCTCAAGTGAT
IL10	ATGGCCGGATAGCCTTATTC	ACGATGTGGTGACAACCGTA
IL12p40	AGTGTGAAGCACCAAATTACTCC	CCCGAGAGTCAGGGGAACT
IL-13	GCAACATCACACAAGACCAGA	GTCAAGGAATCCAGGGCTAC
iNOS	GTTCTCAGCCCAACAATACAAGA	GTGGACGGGTCGATGTCAC
MRC1	CTCTGTTCAGCTATTGGACGC	CGGAATTTCTGGGATTCAGCTTC
TREM2	GAACCGTCACCATCACTCTGA	CCTCGAAACTCGATGACTCCT
Ym1	AGAAGGGAGTTTCAAACCTGGT	CTCTTGCTGATGTGTGTAAGTGA
IFNγR1	GTGGAGCTTTGACGAGCACT	ATTCCCAGCATACGACAGGGT
IL4Rα	ATTTTGCTGTTGGTGACTGGA	CGTGGAAGTGCGGATGTAGT
IL10R	CCTATCCCAAACCAGTCTGAGA	CCAGGTTGAGTTTCCGTACTGT
IL13Rα1	TCAGCCACCTGTGACGAATTT	TGAGAGTGCAATTTGGACTGG
*Scavenger receptors*		
Clec7a	ATTTTGGCGACACAATTCAGGG	GCAAGACTGAGAAAAACCTCCT
Rage	ATGCACAGAAACGGGATCTTT	CTGCTTGGAATAGACACTCCG
*Housekeeping genes*		
ACTB	GCTTCTAGGCGGACTGTTACTGA	GCCATGCCAATGTTGTCTCTTAT
B2M	ATGCACGCAGAAAGAAATAGCAA	AGCTATCTAGGATATTTCCAATTTTTGAA
HMBS	GAAACTCTGCTTCGCTGCATT	TGCCCATCTTTCATCACTGTATG
RPL13a	CCTGCTGCTCTCAAGGTTGTT	TGGTTGTCACTGCCTGGTACTT
TBP	TCTACCGTGAATCTTGGCTGTAAA	TTCTCATGATGACTGCAGCAAA

### Cell isolation

As a control for the western blot analysis, peritoneal macrophages were stimulated to induce the M1 or M2 expression profile. Peritoneal macrophages were isolated from adult mice that were injected with 3 % thioglycollate [25]. On day 4 after injection, mice were euthanized and the peritoneal cavity rinsed with ice-cold PBS. Macrophages were collected and resuspended in RPMI with 1 % FCS. Erythrocytes stayed in suspension and were removed after 45 min. The remaining cells were kept in RPMI with 10 % FCS. The next day, dendritic cells in suspension were removed and macrophages were kept in culture in RPMI with 10 % FCS. Macrophages were stimulated with either LPS (Sigma; 100 ng ml^-1^) and IFNγ (R&D Systems; 100 ng ml^-1^) or IL-4 (R&D Systems; 10 ng ml^-1^) and IL-13 (R&D Systems; 10 ng ml^-1^) to generate M1 or M2 macrophages, respectively.

### Western blot analysis

For western blot analysis, the distal part of the transected *N. ischiadicus* and the contralateral control side were carefully removed; snap frozen and stored at −80 °C until use. Protein lysates were prepared in E1A lysis buffer (1 % NP-40, 20 mM HEPES (pH 7.9), 250 mM NaCl, 20 mM β-glycerophosphate, 10 mM NaF, 1 mM sodium orthovanadate, 2 mM dithiothreitol, 1 mM EDTA, and a protease inhibitor cocktail) by homogenization in a Potter Elvehjem homogenisator. Total protein concentration was determined by Bradford. Equal amounts of protein lysates (20 to 30 μgram) were separated on NuPAGE gels, transferred to nitrocellulose membranes and analyzed by immunoblotting. Briefly, membranes were blocked using blocking buffer (5 % milk in PBS containing 0.1 % Tween-20), and incubated overnight at 4 °C with a primary antibody. Secondary HRP-conjugated antibodies were used to visualize antibody signals on films using the ECL system (Thermo Scientific). Antibodies used were anti-arginase-1 (Santa Cruz; 1/1,000), anti-iNOS (BD Transduction Laboratories; 1/1,000) and anti-β-tubulin (Abcam; 1/10,000).

### Immunohistochemistry

The dissected distal part of the sciatic nerve was fixed overnight in Bouin’s fixative (saturated picric acid, formaldehyde, and glacial acetic acid). The nerves were dehydrated, embedded in paraffin for longitudinal sectioning, and stored until processing. The 4 μm sections were immunohistochemically stained using the avidin-biotin staining technique. Briefly, slides were rehydrated and antigen retrieval was performed by boiling the slides in a citrate buffer (0.1 M sodium citrate buffer; pH 6.0). Endogenous peroxidase activity was blocked by treating the slides with 2 % hydrogen peroxide for 20 min. Next, the slides were incubated for 30 min in blocking buffer (20 % normal goat serum in PBS with 1 % BSA) and incubated overnight with primary antibody at 4 °C. The antibodies used were arginase-1 (Santa Cruz) and iNOS (BD Transduction Laboratories). Sections were treated with avidin-conjugated secondary antibodies for 30 min at room temperature before adding an avidin-biotin complex solution (Vector Laboratories) for 30 min. The signal was visualized by incubating the sections with 3.3’-diaminobenzidine (DAB) in PBS containing 0.1 % hydrogen peroxide. Negative control sections were handled the same way, but in the absence of primary antibody. All sections were counterstained with hematoxylin and dehydrated before mounting. Sections were analyzed with a standard light microscope (Zeiss Axioskop with Olympus UC30 camera). For double immunofluorescence staining, the 4 μm sections of paraffin-embedded sciatic nerves, which were processed for antigen retrieval as described above, were incubated in a blocking solution (1 % milk in PBS) for 30 min and incubated at 4 °C with goat-anti-arginase-1 antibody (Santa Cruz). The next day, a donkey-anti-goat Alexa fluor 488 conjugated secondary antibody (Life Technologies) was applied. After stringent washing, the staining with the second marker was performed with the same procedure, using a marker for macrophages (F4/80; Santa Cruz) and a marker for Schwann cells (S100; Dako) and an Alexa fluor 594 conjugated secondary antibody (Life Technologies). Negative controls, excluding one or both of the primary antibodies, were included in the experiments. The immunofluorescence images were captured on a Zeiss LSM700 confocal microscope, using a 40× (1.3 NA) objective. Frame-by-frame scanning with standard emission settings and excitation with a 488 nm or 555 nm diode laser was used to discriminate the two fluorophores.

## Results

**Figure 1:**
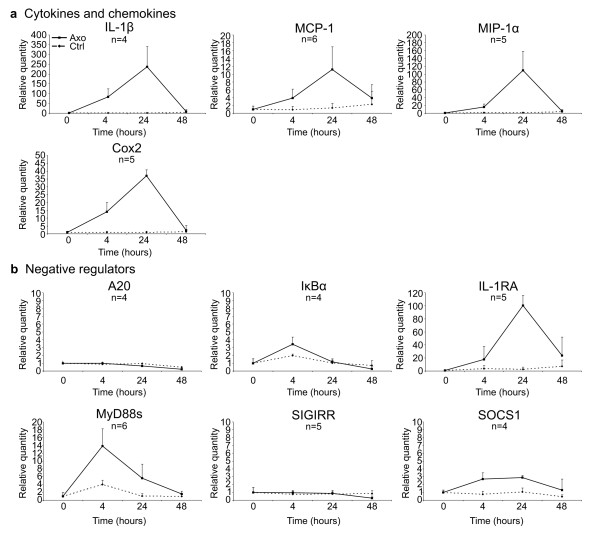
**Peripheral nerve injury triggers a transient immune response.** RT-qPCR analysis showing the transient induction of immune mediators at different time points after peripheral nerve injury (**a**), and the induction of negative regulators of the innate immune response (**b**), as determined by RT-qPCR analysis. RT-qPCR data are analyzed using the ΔΔ-Ct method and mRNA expression levels are expressed relative to the basal condition (=0 h time point). Data presented are the compiled data of different independent experiments, with n representing the number of independent experiments for each gene.

Wallerian degeneration (WD) induces an immune response that is considered to be predominantly pro-inflammatory by expressing several pro-inflammatory molecules such as TNF, IFNγ, and iNOS [19-21]. To confirm the pro-inflammatory environment, we isolated total RNA of the distal segment of four sciatic nerves isolated at different time points upon axotomy. We analyzed three independent experiments and measured the expression of several cytokine and chemokine transcripts using RT-qPCR. In line with literature data, the inflammatory mediators IL-1β, Cox2, MCP-1, and MIP-1α were strongly up-regulated, with maximum expression levels at 24 h after axotomy (Figure 1a) [4]. Strikingly, the expression levels of the inflammatory genes dropped at later time points after axotomy, with most pro-inflammatory genes returning to the basal condition at 48 h. We determined whether this transient immune response was accompanied with the induction of several negative regulators of the immune system and found that IL-1RA displayed a high induction (Figure 1b). Moreover, MyD88small (MyD88s) and IκBα, both negatively regulating NF-κB activation [26], were up-regulated already 4 h after injury. In contrast, two other negative regulators of the NF-κB pathway, A20 and SIGIRR, were not induced. Suppressor of cytokine signaling 1 (SOCS1) was only weakly induced after axotomy at these early time points (Figure 1b). Functions of the immune mediators and negative regulators are shown in Table 2.

**Table 2:** Overview of the main functions of immune mediators and negative regulators of the immune system

**Immune mediators**		
MCP-1 (CCL2)	Monocyte chemoattractant protein- 1 (CC-chemokine 2)	Chemokine, emigration of monocytes from bone marrow; monocyte recruitment [27]
COX2	Cyclo-oxygenase 2	Production of prostaglandins; vasodilatation, inflammation, platelet disaggregation [28], neuropathic pain [29]
IL-1β	Interleukin-1 beta	Pro-inflammatory cytokine; many biological functions: up-regulation of adhesion molecules, influx of neutrophils, induction of additional inflammatory mediators, important in sterile inflammation [30]
IL-6	Interleukin 6	Pleiotropic cytokine; wide range of biological activities in immune regulation, hematopoiesis, and inflammation [31]
MIP-1α (CCL3)	Macrophage inflammatory protein 1 alpha (CC-chemokine 3)	Chemokine, recruitment of monocytes to the inflamed tissue [4]
**Negative regulators**		
A20	-	Negative regulator of TLR-signaling. Blocks TLR-mediated signaling by blocking NFκB signaling [32]
IκBα	Inhibitor kappa B alpha	Inhibits NFκB signaling by masking the nuclear localization signal, keeping NFκB in its inactive state [33]
IL-1RA	IL-1 receptor antagonist	Antagonist of IL-1; binds to IL-1R1 thereby blocking signaling [30]
MyD88s	Myeloid differentiation 88 small	Antagonist of the adaptor protein MyD88 [26]
SIGIRR	Single immunoglobulin IL-1R-related molecule	Inhibits IL-1 signaling, orphan receptor of IL-1 family with antagonistic properties [34]
SOCS1	Suppressor of cytokine signaling 1	Suppression of cytokine signaling by inhibiting JAK tyrosine kinase [35]

**Figure 2:**
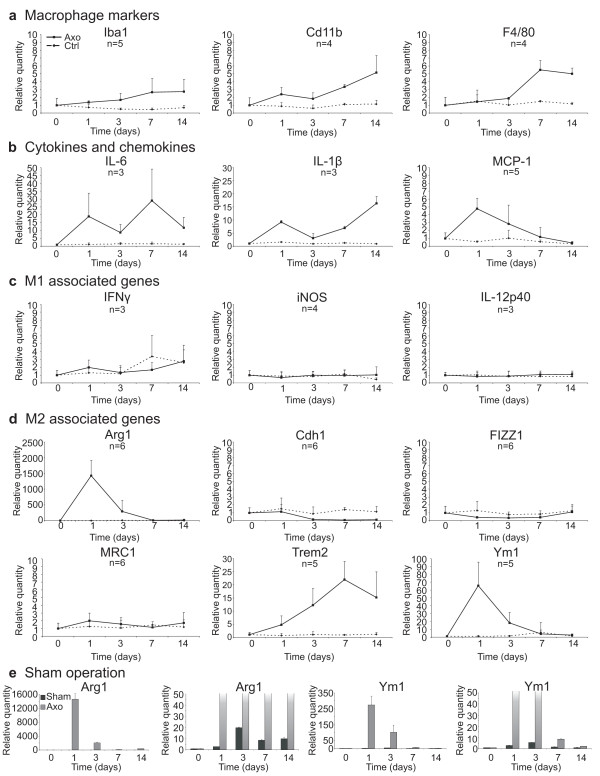
**Peripheral nerve injury induces M2 associated genes.** RT-qPCR analysis of the immune response up to 14 days post injury, showing macrophage markers (**a**), cytokine expression (**b**), M1 associated genes **(c)**, and M2 associated markers (**d**). Sham operation induces M2 markers, but only to a minor extent compared to acute neurodegeneration (**e**). The mRNA levels are expressed relative to the basal condition (=0 h time point). Data presented are the compiled data of different independent experiments, with n representing the number of independent experiments for each gene. For sham operation one representative experiment out of two independent experiments is shown.

**Figure 3:**
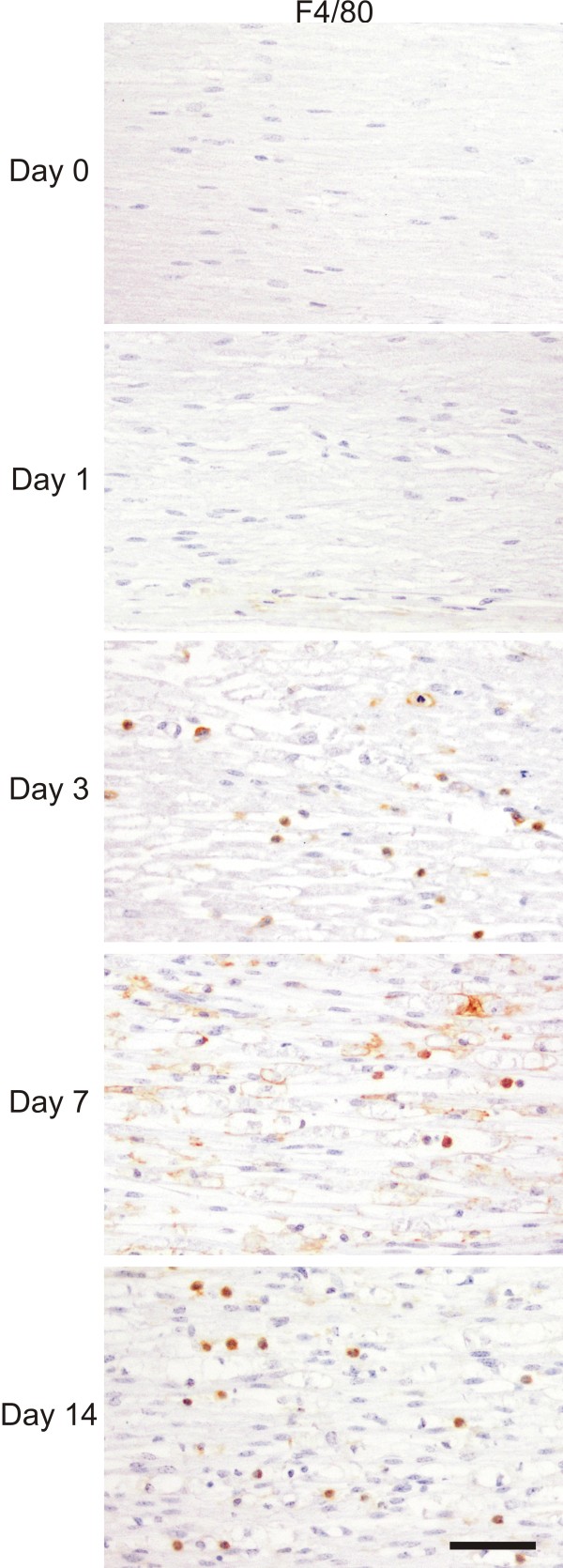
**Accumulation of macrophages in the injured sciatic nerve.** Immunohistochemical staining of sciatic nerve slices for F4/80 at different time points after injury. Scale bar 50 μm.

**Figure 4:**
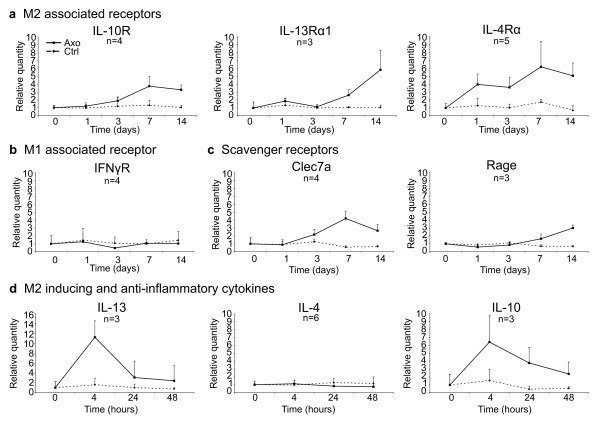
**Peripheral nerve injury is associated with the expression of M2-associated cytokines and cytokine receptors.** RT-qPCR analysis of the sciatic nerve showing cytokine receptor expression profiles upon injury (**a, b**), scavenger receptor expression (**c**), and expression of M2 cytokines (**d**). The mRNA levels are expressed relative to the basal condition (=0 h time point). Data presented are the compiled data of different independent experiments, with n representing the number of independent experiments for each gene.

While many reports already described the induction of cytokines and chemokines in WD, it is less obvious what type of immune response is triggered by injury in the PNS. Therefore, we decided to focus on gene expression profiles for genes associated with M1 *vs.* M2 macrophages, representative for the two extremes of a merely pro-inflammatory *vs.* a merely anti-inflammatory/wound healing phenotype [25,36,37]. The main functions of these genes are described in Table 3. We first determined when macrophages start to accumulate in our model, by analyzing the presence of three universal markers for macrophages (Iba1, CD11b, and F4/80) using RT-qPCR [38,39]. In general, it is considered that a first contribution to the immune response in the nerve is mediated by resident cells (such as Schwann cells, resident macrophages, and fibroblasts) because blood-borne monocytes infiltrate the nerve only 2 to 3 days after injury [12]. Macrophages, expressing Iba1, CD11b, and F4/80, start to accumulate in the injured nerves from day 3 onwards as determined by RT-qPCR (Figure 2a) and immunohistochemistry (Figure 3). Coinciding with the accumulation of macrophages, a second peak in the immune response could be observed, as shown by the biphasic induction of IL-6 and IL-1β expression (Figure 2b). As expected, MCP-1, a chemoattractant for macrophages produced by Schwann cells, is expressed right before macrophage accumulation (Figure 2b). In order to determine the phenotype of the macrophages present in the peripheral nerve after injury, we analyzed markers typically associated with M1 *vs.* M2 macrophages. None of the M1 markers such as iNOS, IL-12p40, and IFNγ were induced after axotomy at any time point investigated (Figure 2c). On the other hand, the M2-associated genes, arginase-1 and Ym1, were clearly induced. The expression of these genes reached a maximum at 1 day after axotomy and returned to basal level at day 7. Another typical marker for M2 macrophages, Trem2, was induced from day 3 onwards and its expression level remained elevated till day 14 after axotomy (the latest time point investigated in this study) (Figure 2d). The expression of Trem2 appeared to be mediated by the accumulating macrophages, as its expression level displayed a similar pattern as the general macrophage markers (Figure 2b and d). Some markers (arginase-1 and Ym1) were also slightly induced in sham-operated animals, however this induction was only minor compared to the induction seen after axotomy (Figure 2e). Altogether, these data suggest that acute peripheral nerve injury favors an M2 macrophage environment. Additional analyses confirmed this hypothesis. We found that receptors known to trigger M2 cells (such as IL-4Rα and IL-13Rα1), and to stimulate macrophage suppressor function (such as IL-10R), were induced in injured peripheral nerves at 7 and 14 days after injury (Figure 4a). The IFNγR1 receptor, which characterizes M1 macrophages, was not enhanced (Figure 4b). Moreover, scavenger receptors (such as Clec7a and Rage), which are typically expressed by M2 macrophages, showed an increased expression level after axotomy at the late time points relative to the uninjured control nerve (Figure 4c).

**Table 3:** Overview of the main functions of M1 and M2 associated markers

**General macrophage markers**		
CD11b	Cluster of differentiation 11b	Expressed on all myeloid lineage cells
F4/80 (EMR1)	EGF-like module-containing mucin-like hormone receptor-like 1	Expressed on most tissue macrophages
Iba1	Ionized calcium binding adaptor molecule 1	Expressed on macrophages (and microglia)
**M1 associated markers**		
IFNγ	Interferon gamma	Polarize macrophages towards M1; critical for innate and adaptive immunity against viral and intracellular bacterial infections (also produced by macrophages) [40]
IL-12p40	Interleukin 12 subunit p40	Pro-inflammatory cytokine; induces Th1[41], production of cytokines (mainly IFNγ) [42], stimulation cell-mediated immunity against microbial pathogens [43]
iNOS	Inducible nitric oxide synthase	Production of nitric oxide (NO) using arginine as a substrate; defense against microorganisms [44]
**M2 associated markers**		
Arg1	Arginase 1	Production of L-ornithine and urea using arginine as a substrate, first step to production of polyamines (growth factors in the nervous system) [45]; counteracts iNOS
CDH1	Cadherin-1	Cell-cell adhesion [46], interaction with CD103 of regulatory T-cells [47]
Clec7a (dectin1)	C-type lectin domain family 7, member a	Pattern recognition; non-opsonic beta-glucan receptor [48]
FIZZ1	Found in inflammatory zone	Lipid and sugar metabolism; angiogenesis-promoting factor [49]
IL-4	Interleukin 4	Th2 effector cytokine: polarize macrophages to M2 [50]; IgE class switching, differentiation of CD4+ T-cells into Th2 cells [51]
IL-10	Interleukin 10	Anti-inflammatory cytokine [52]
IL-13	Interleukin 13	Th2 effector cytokine: polarize macrophages to M2; fibrosis; defense against parasite infections [53]
MRC1	Mannose receptor type C1	Pattern recognition, type II response promoting effect [54]
Rage	Receptor for advanced glycosylation end products	Pattern recognition; binds endogenous ligands such as S100B to stimulate trophic effects on neurons [55]
Trem2	Triggering receptor expressed on myeloid cells 2	Pattern recognition [56]; augmentation of apoptotic neuron phagocytosis and attenuation of proinflammatory cytokine secretion [57]
Ym1 (CH3L3)	Chitinase 3-like-3	Weak eosinophils chemoattractant properties [58]; binds heparan sulfate, heparin, GlcN olimers; Th2 promoting effect [59]

The M2 gene expression profile is typically triggered by the cytokines IL-4 and/or IL-13 [60]. In order to determine if these cytokines play a role in the induction of the alternative macrophage environment after axotomy, their expression level was investigated at early time points using RT-qPCR. The IL-4 expression was hardly detectable at the mRNA level in our model of acute peripheral nerve injury and did not seem to be induced. The IL-13 expression, however, was induced upon axotomy at the earliest time point (4 h) investigated (Figure 4d). Importantly, also the anti-inflammatory cytokine IL-10 was induced after injury (Figure 4d). The high IL-10 and low IL-12p40 expression levels are representative of a typical M2 activation profile (Figures 2c and 4d).

**Figure 5:**
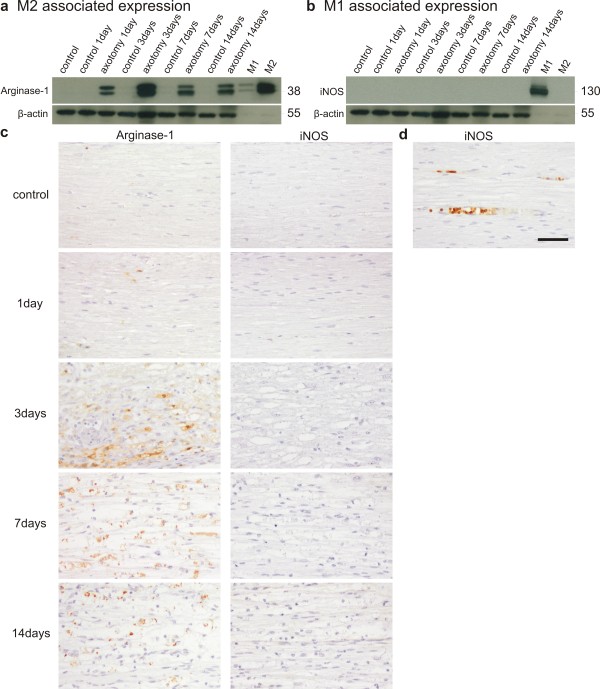
**Protein analysis of M1 versus M2 markers after PNS injury.** Western blot analysis showing the arginase-1 protein induction in axotomized sciatic nerves. The IL-4/IL-13 stimulated peritoneal macrophages were used as a positive control (M2) (**a**). Western blot analysis showing iNOS protein induction in axotomized sciatic nerves. Peritoneal macrophages stimulated with IFNγ/LPS served as a positive control (M1) (**b**). For western blot analysis, MW values expressed in kDa are shown at the right side of the blots. Immunohistochemical staining of sciatic nerve slices for arginase-1 and iNOS at different time points after injury (**c**). Blood vessels in the nerves stain positive for iNOS by immunohistochemical staining (**d**). Scale bar 50 μm.

**Figure 6:**
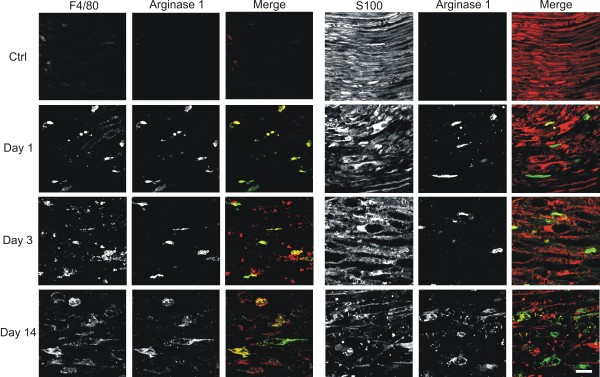
**Arginase-1 positive cells in the injured peripheral nerve tissue are macrophages.** Double immunofluorescently stained peripheral nerve sections at day 0, day 1, day 3, and day 14 upon axotomy showing arginase-1 colocalizing with F4/80, a general macrophage marker. F4/80, red; S100, red; arginase-1, green. Scale bar 20 μm.

Next we analyzed the macrophage phenotype at protein level by using western blot and immunohistochemistry. As the balance between arginase-1 and iNOS expression is highly indicative of the macrophage phenotype, these two markers were used in the following experiments [36]. Western blot analysis of protein lysates of the distal segment of the sciatic nerve showed an induction of arginase-1 protein after axotomy. Arginase-1 protein was detectable from day 1 after injury and reached a maximal signal at day 3. Albeit showing a small decrease over time, the arginase-1 protein level remained high until day 14 after axotomy (Figure 5a). iNOS was not detectable at any time point by western blot analysis, confirming our RT-qPCR data (Figure 5b). As a positive control, peritoneal macrophages were stimulated *in vitro* with either IL-4/IL-13 or LPS/IFNγ to obtain M2 and M1 macrophages, respectively. As expected, the M2 macrophages expressed arginase-1 and the M1 macrophages expressed iNOS protein (Figure 5a, b). Immunohistochemistry of paraffin-embedded sciatic nerves confirmed the temporal expression profile for arginase-1 shown by western blot. Arginase-1 is rapidly expressed throughout the entire injured nerve. The expression level peaked at 3 days post injury and remained high until day 14 (Figure 5c). Double immunofluorescence staining revealed that arginase-1 was present in F4/80 positive cells and not in S100 positive Schwann cells, which identifies macrophages as the main source for arginase-1 (Figure 6). While at earlier time points all cells that expressed F4/80 were found to be positive for arginase-1, at later time points arginase-1 negative macrophages were present as well. Immunohistochemical staining for iNOS confirmed that this protein was not induced after axotomy (Figure 5c). We only observed strong iNOS staining in blood capillaries in particular regions on the nerve that was present independently of the axotomy, showing that the antibody staining was working properly (Figure 5d).

**Figure 7:**
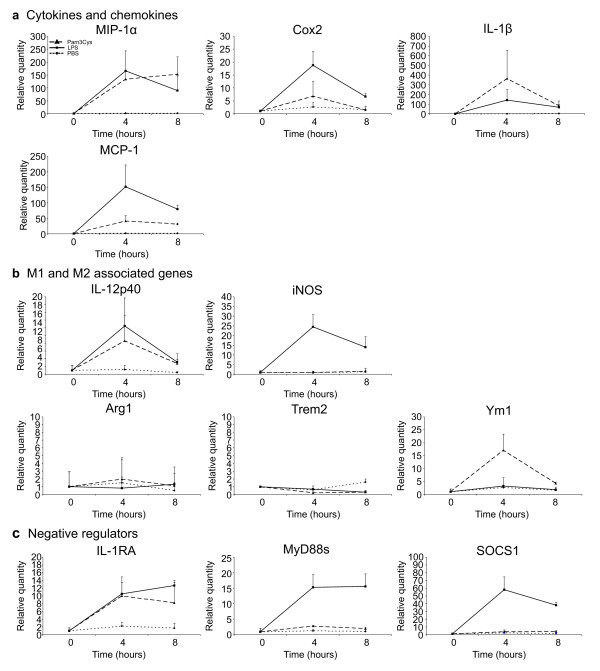
**Immune response in the sciatic nerve of mice intravenously injected with TLRligands.** Cytokine and chemokine induction in the sciatic nerves after intravenous injection of LPS (TLR4 ligand) or Pam3Cys (TLR1/2 ligand). PBS injection was used as a negative control (**a**). M1- and M2-associated gene expression after TLR ligand injection (**b**). Negative regulators are also induced upon LPS and Pam3Cys injection (**c**). Data represented are the compiled data of two independent experiments.

Finally, we determined whether the M2-predominated immune response triggered after nerve injury is typical for the PNS or whether it is specific for neurodegeneration. To this end, we investigated at different time points the expression of M1 and M2 markers in sciatic nerves from mice intravenously injected with TLR-ligands. We used lipopolysaccharide (LPS), a TLR4 ligand known to induce a classical type I immune response, and Pam3Cys, a TLR1/2 ligand. Intravenous injection of LPS as well as Pam3Cys elicited a rapid and strong immune response in the sciatic nerve, as shown by the induction of inflammatory genes such as IL-1β, Cox2, MIP-1α, and MCP-1 (Figure 7a). Interestingly, the pro-inflammatory cytokine IL-12p40 and typical M1 immune mediator iNOS, both representative for a type I immune response, were induced after LPS injection (Figure 7b). Several negative regulators, such as IL-1RA, MyD88s, and SOCS1, which mediate a negative feedback loop, were also induced by LPS injection (Figure 7c). Injection with Pam3Cys, however, clearly induced a mixed immune response as reflected by the expression of the M1 associated cytokine IL-12p40 and the expression of Ym1, which is an M2-associated macrophage marker (Figure 7b). iNOS was not detectable after Pam3Cys injection (Figure 7b) and none of the other M2-associated genes such as arginase-1 and Trem2 were induced (Figure 7b). These data show that a prototypical type I immune response can be observed in the nerve after injection of LPS, while Pam3Cys seems to induce a mixed immune response. Both TLR-mediated responses clearly differed from the immune response induced after acute peripheral nerve injury.

## Discussion

In response to an infection, a strong pro-inflammatory immune response is triggered. The recruited inflammatory cells are activated when they encounter pathogen-associated molecular products (PAMPs) such as LPS. Hereupon, these cells phagocytose infectious agents and produce pro-inflammatory mediators such as iNOS, IL-12, ROS, and RNS to fight off the invading pathogen. These agents, however, can also cause tissue damage. The innate immune system also detects the presence of endogenous molecules; so-called danger associated molecular patterns (DAMPs) that are only exposed in conditions of injury. Under conditions of cellular stress or injury, one might expect a more dampened, strictly controlled immune response as the cost-benefit ratio is higher. As we and others have shown, pro-inflammatory mediators such as IL-1β and Cox2 and chemokines such as MIP-1α and MCP-1 are rapidly induced in WD, a model of sterile inflammation in the nerve [3,4]. In the present study we show that the expression of these inflammatory genes is strictly controlled as the mRNA levels of all cytokines and chemokines return to basal level at 48 h. Negative regulators of the pro-inflammatory signaling pathways are induced prior to the decline in inflammatory gene expression, thereby limiting the pro-inflammatory immune response and also the excessive damage caused by the immune system.

Although PAMPs and DAMPs are recognized by the same set of receptors, such as TLRs, they can induce a different set of genes [61]. Bacterial compounds trigger a microbicidal environment and classically activated M1 macrophages; while endogenous molecules seem to activate an inflammatory response associated with genes that mediate tissue repair [61,62]. Since most studies so far focused specifically on the induction of pro-inflammatory mediators, WD in the PNS has always been associated with the induction of a strong pro-inflammatory immune response. We found, however, by analyzing genes associated with M1 and M2 macrophages, that acute peripheral nerve injury rather induces an M2-like macrophage environment. None of the typical pro-inflammatory markers of the M1 subtype of macrophages such as iNOS, IFNγ, and IL-12p40 could be detected, while M2 markers such as arginase-1, Ym1, and Trem2 were highly up-regulated. Intriguingly, other M2 markers like Fizz1 and Cdh1 were not induced. Van den Bossche *et al*. showed that some M2 markers like Cdh1 are strongly down-regulated by the presence of pro-inflammatory cytokines [63]. This could be the case here as well. The stimulation of the alternative macrophage environment in the nerve appeared to be controlled at the level of IL-13. This cytokine was readily detectable from 4 h after the onset of neurodegeneration, and prior to the expression of arginase-1 and Ym1. IL-13, which is together with IL-4 a central master switch in the M2 phenotype, is typically expressed by macrophages, basophils, mast cells, or activated T cells [64]. Since we detect accumulation of macrophages only from days 2 to 3 onwards, it is less clear at the moment which cells are responsible for the early onset expression of IL-13, arginase-1, or Ym1. In the peripheral nerve resident macrophages, mast cells or SCs could be engaged in the expression of IL-13, while neutrophils could contribute to the expression of arginase-1 and Ym1. Neutrophils are recruited to the damaged nerves at day 1 after injury [65], and are suggested to contribute to the expression of tissue repair genes [66]. Our results demonstrate that damage to the nerve establishes a rapid immunosuppressive reaction within the nerve, and this from very early time points on, which appears to be in contrast with another recent report [67]. Shechter *et al*. described that axotomy of the optic nerve creates a pro-inflammatory environment in the nerve that was later turned into an anti-inflammatory one by infiltrating macrophages [67]. Macrophages have been shown before to play a beneficial role in WD in the PNS, as depleting them impaired functional recovery [68]. By phagocytosing debris, macrophages contribute to regeneration by removing inhibitory myelin debris and paving the way for neurite outgrowth. Pre-existing auto-antibodies have been shown to play an important role in clearance of myelin debris by promoting a macrophage influx and stimulating their phagocytic activity [69]. In addition, macrophages produce neurotrophic factors, thereby supporting regeneration [70]. The protective role of macrophages in WD might also be explained by their phenotype. The M2 macrophages were shown to be neuroprotective *in vitro* by stimulating neurite outgrowth, while M1 macrophages were neurotoxic to neuronal cell cultures [71]. Moreover, potent inducers of a systemic Th2 switch, such as glatiramer acetate and statins, support the neuroprotection and/or nerve regeneration [72-74]. The Th2-inducing adjuvants, such as IFA and Alum, promote axon regeneration better than the Th1-inducing adjuvant CFA [75,76]. Also Th2 cells support neuronal survival *in vitro* to a greater extent than Th1 cells [77].

In autoimmune diseases of the PNS such as Guillian-Barré Syndrome (GBS) and chronic inflammatory demyelinating polyneuropathy (CIDP), a Th1 response is associated with the early stages of the disease. During recovery of GBS and CIDP, a shift towards a Th2 response is observed, suggesting a protective role for Th2 responses in these diseases [78-80]. Also from animal models it is apparent that type II immune responses are beneficial; as nasal administration of recombinant IL-4 ameliorates ongoing experimental autoimmune neuritis (EAN) and inhibits demyelination [81]. The self-limiting clinical course of GBS might be explained by the induction of IL-4 and IL-10. The role of the immune system in hereditary neuropathies is less well studied. Patients suffering from inherited neuropathies show endoneurial T-cells in their nerve biopsies and some patients even show inflammatory infiltrates [82,83]. Studies with animal models such as the heterozygote P0 mice, a model of Charcot-Marie-Tooth (CMT) 1B neuropathy, clearly show a functional degenerative role for macrophages and T-cells [84,85]. Unfortunately, the type of immune response triggered in hereditary neuropathies has not been addressed.

In CNS injury, macrophages have been implicated in both exacerbating as well as ameliorating tissue damage at the injury site. Kigerl *et al*. showed that spinal cord injury (SCI) initially induces both M1 and M2 macrophages, however, the M1 phenotype predominates the lesion site after 1 week [71]. The presence of both phenotypes might explain the dual effect of macrophages in this model. Moreover, axonal regeneration after SCI is prevented by an inhibitory environment due to myelin inhibitors. Qui *et al*. showed that elevating cAMP was sufficient to overcome the myelin-mediated inhibition [86]. Subsequent studies showed that arginase-1 and polyamines played a major protective role downstream of cAMP [45]. By using PNS grafts together with acidic fibroblast growth factor in a model of SCI, the recruited macrophages produced large amounts of arginase-1 and were involved in polyamine synthesis [87]. This strategy significantly improved functional recovery [88]. Altogether these data show a central role for type II immune responses, arginase-1 and downstream polyamines in regeneration.

Lessons from graft implantation in CNS injury showed that PNS tissue induces a permissive environment for regeneration. Macrophages contribute to this permissive environment as spinal cord-injured axons failed to regenerate through peripheral nerve grafts in the absence of CD11b^+^ cells [68]. We now hypothesize that PNS injury triggers an inherent protective environment by inducing an M2 phenotype of macrophages and arginase-1 expression. This model can further be used to unravel how the environment is induced and to elucidate which protective program needs to be elicited. Currently, it remains to be shown how the alternative macrophage environment is established; however there might be a role for IL-13, a typical M2 inducer, as this cytokine is up-regulated very early after injury and prior to the M2-associated gene expression.

Finally, the induction of the alternative macrophage environment appeared to be triggered specifically in response to neurodegeneration. Our results show that when challenged with bacterial products such as LPS, a typical pro-inflammatory immune response, as reflected by a strong IL-12p40 and iNOS signal and the absence of Ym1 or arginase-1, could be detected within the PNS. Intriguingly, injection with Pam3Cys, a TLR1/2 ligand, induced a mixed response marked by presence of both IL-12p40 and Ym1 induction. TLR2, the co-receptor for TLR1, has been associated before with the induction of a type II gene expression [89,90]. In a recent study we showed that specifically TLR1 was highly induced after acute peripheral nerve injury and hypothesized that it might play a role in detecting neuronal injury [22]. The possible involvement of TLR1/2 in the detection of peripheral nerve injury and in the switch towards the type II gene expression is currently under investigation.

## Conclusion

In conclusion, we demonstrate that acute peripheral nerve injury induces an inherent protective response with the initiation of several negative feedback loops, limiting excessive tissue damage. Furthermore, we show that an M2-like anti-inflammatory environment is induced, rather than a pro-inflammatory one. Since type II responses have been shown before to be neuroprotective, we believe that instead of inhibiting the immune responses, shifting the macrophage phenotype or type of immune response towards an alternative activation state or type II response would be a better therapeutic strategy to stimulate repair, as this would create a permissive environment for neuronal regeneration.

## Abbreviations

CFA, Complete freund’s adjuvant; CIDP, Chronic inflammatory demyelinating polyneuropathy; CMT, Charcot-Marie-Tooth; CNS, Central nervous system; DAB, 3,3'-Diaminobenzidine; DAMP, Danger associated molecular pattern; EAN, Experimental autoimmune neuritis; GBS, Guillian-Barré Syndrome; IFA, Incomplete freund’s adjuvant; LPS, Lipopolysaccharide; MyD88s, Myeloid differention 88 small; PAMP, Pathogen associated molecular pattern; PNS, Peripheral nervous system; RNS, Reactive nitric species; ROS, Reactive oxygen species; RT-qPCR, Real-time quantitative polymerase chain reaction; SC, Schwann cell; SCI, Spinal cord injury; SOCS1, Suppressor of cytokine signaling 1; WD, Wallerian degeneration.

## Competing interests

The authors declare that they have no competing interests.

## Authors’ contributions

EY carried out the experiments and wrote the manuscript, together with SJ. AC preformed the intravenous injections. BA provided expertise on microscopy. SG, LP, and GL assisted in the animal surgery and experiments. LAS and JAVG contributed to the interpretation of the results and discussed analyses. VT and SJ designed the methods and experiments. All the authors read and approve the final version of the manuscript.

## References

[B1] Waller A Experiments on the section of the glossopharyngeal and hypoglossal nerves of the frog, and observations of the alterations produced thereby in the structure of their primitive fibres Phil Transact Royal Soc London 1850 140 423 429 10.1098/rstl.1850.0021

[B2] Saxena S Caroni P Mechanisms of axon degeneration: from development to disease Prog Neurobiol 2007 83 174 191 10.1016/j.pneurobio.2007.07.007 17822833

[B3] Shamash S Reichert F Rotshenker S The cytokine network of Wallerian degeneration: tumor necrosis factor-alpha, interleukin-1alpha, and interleukin-1beta J Neurosci 2002 22 3052 3060 11943808 10.1523/JNEUROSCI.22-08-03052.2002 PMC6757534

[B4] Perrin FE Lacroix S Aviles-Trigueros M David S Involvement of monocyte chemoattractant protein-1, macrophage inflammatory protein-1alpha and interleukin-1beta in Wallerian degeneration Brain 2005 128 854 866 10.1093/brain/awh407 15689362

[B5] Taskinen HS Olsson T Bucht A Khademi M Svelander L Roytta M Peripheral nerve injury induces endoneurial expression of IFN-gamma, IL-10 and TNF-alpha mRNA J Neuroimmunol 2000 102 17 25 10.1016/S0165-5728(99)00154-X 10626662

[B6] Subang MC Richardson PM Influence of injury and cytokines on synthesis of monocyte chemoattractant protein-1 mRNA in peripheral nervous tissue Eur J Neurosci 2001 13 521 528 10.1046/j.1460-9568.2001.01425.x 11168559

[B7] Taskinen HS Roytta M Increased expression of chemokines (MCP-1, MIP-1alpha, RANTES) after peripheral nerve transection J Peripher Nerv Syst 2000 5 75 81 10.1046/j.1529-8027.2000.00009.x 10905466

[B8] Toews AD Barrett C Morell P Monocyte chemoattractant protein 1 is responsible for macrophage recruitment following injury to sciatic nerve J Neurosci Res 1998 53 260 267 10.1002/(SICI)1097-4547(19980715)53:2<260::AID-JNR15>3.0.CO;2-A 9671983

[B9] Zuo Y Perkins NM Tracey DJ Geczy CL Inflammation and hyperalgesia induced by nerve injury in the rat: a key role of mast cells Pain 2003 105 467 479 10.1016/S0304-3959(03)00261-6 14527707

[B10] Moalem G Tracey DJ Immune and inflammatory mechanisms in neuropathic pain Brain Res Rev 2006 51 240 264 10.1016/j.brainresrev.2005.11.004 16388853

[B11] Bendszus M Stoll G Caught in the act: in vivo mapping of macrophage infiltration in nerve injury by magnetic resonance imaging J Neurosci 2003 23 10892 10896 14645484 10.1523/JNEUROSCI.23-34-10892.2003 PMC6740995

[B12] Mueller M Leonhard C Wacker K Ringelstein EB Okabe M Hickey WF Kiefer R Macrophage response to peripheral nerve injury: the quantitative contribution of resident and hematogenous macrophages Lab Invest 2003 83 175 185 12594233 10.1097/01.lab.0000056993.28149.bf

[B13] Taskinen HS Roytta M The dynamics of macrophage recruitment after nerve transection Acta Neuropathol 1997 93 252 259 10.1007/s004010050611 9083556

[B14] Perry VH Brown MC Gordon S The macrophage response to central and peripheral nerve injury. A possible role for macrophages in regeneration J Exp Med 1987 165 1218 1223 10.1084/jem.165.4.1218 3559478 PMC2188570

[B15] Mueller M Wacker K Ringelstein EB Hickey WF Imai Y Kiefer R Rapid response of identified resident endoneurial macrophages to nerve injury Am J Pathol 2001 159 2187 2197 10.1016/S0002-9440(10)63070-2 11733369 PMC1850587

[B16] Hirata K Kawabuchi M Myelin phagocytosis by macrophages and nonmacrophages during Wallerian degeneration Microsc Res Tech 2002 57 541 547 10.1002/jemt.10108 12112437

[B17] Omura T Omura K Sano M Sawada T Hasegawa T Nagano A Spatiotemporal quantification of recruit and resident macrophages after crush nerve injury utilizing immunohistochemistry Brain Res 2005 1057 29 36 10.1016/j.brainres.2005.07.008 16112089

[B18] Sawada T Sano M Omura T Omura K Hasegawa T Funahashi S Nagano A Spatiotemporal quantification of tumor necrosis factor-alpha and interleukin-10 after crush injury in rat sciatic nerve utilizing immunohistochemistry Neurosci Lett 2007 417 55 60 10.1016/j.neulet.2007.02.028 17336456

[B19] Liefner M Siebert H Sachse T Michel U Kollias G Bruck W The role of TNF-alpha during Wallerian degeneration J Neuroimmunol 2000 108 147 152 10.1016/S0165-5728(00)00262-9 10900348

[B20] Gillen C Jander S Stoll G Sequential expression of mRNA for proinflammatory cytokines and interleukin-10 in the rat peripheral nervous system: comparison between immune-mediated demyelination and Wallerian degeneration J Neurosci Res 1998 51 489 496 10.1002/(SICI)1097-4547(19980215)51:4<489::AID-JNR8>3.0.CO;2-8 9514202

[B21] de la Hoz CL Oliveira AL Queiroz Lde S Langone F Wallerian degeneration in C57BL/6J and A/J mice: differences in time course of neurofilament and myelin breakdown, macrophage recruitment and iNOS expression J Anat 2003 203 567 578 10.1046/j.1469-7580.2003.00248.x 14686692 PMC1571200

[B22] Goethals S Ydens E Timmerman V Janssens S Toll-like receptor expression in the peripheral nerve Glia 2010 58 1701 1709 10.1002/glia.21041 20578041

[B23] Vandesompele J De Preter K Pattyn F Poppe B Van Roy N De Paepe A Speleman F Accurate normalization of real-time quantitative RT-PCR data by geometric averaging of multiple internal control genes Genome Biol 2002 3 RESEARCH0034 12184808 10.1186/gb-2002-3-7-research0034 PMC126239

[B24] Hellemans J Mortier G De Paepe A Speleman F Vandesompele J qBase relative quantification framework and software for management and automated analysis of real-time quantitative PCR data Genome Biol 2007 8 R19 10.1186/gb-2007-8-2-r19 17291332 PMC1852402

[B25] Ghassabeh GH De Baetselier P Brys L Noel W Van Ginderachter JA Meerschaut S Beschin A Brombacher F Raes G Identification of a common gene signature for type II cytokine-associated myeloid cells elicited in vivo in different pathologic conditions Blood 2006 108 575 583 10.1182/blood-2005-04-1485 16556895

[B26] Janssens S Burns K Tschopp J Beyaert R Regulation of interleukin-1- and lipopolysaccharide-induced NF-kappaB activation by alternative splicing of MyD88 Curr Biol 2002 12 467 471 10.1016/S0960-9822(02)00712-1 11909531

[B27] Tsou CL Peters W Si Y Slaymaker S Aslanian AM Weisberg SP Mack M Charo IF Critical roles for CCR2 and MCP-3 in monocyte mobilization from bone marrow and recruitment to inflammatory sites J Clin Invest 2007 117 902 909 10.1172/JCI29919 17364026 PMC1810572

[B28] Morita I Distinct functions of COX-1 and COX-2 Prostaglandins Other Lipid Mediat 2002 68-69 165 175 12432916 10.1016/s0090-6980(02)00029-1

[B29] Ma W Quirion R Does COX2-dependent PGE2 play a role in neuropathic pain? Neurosci Lett 2008 437 165 169 10.1016/j.neulet.2008.02.072 18434017

[B30] Dinarello CA Immunological and inflammatory functions of the interleukin-1 family Annu Rev Immunol 2009 27 519 550 10.1146/annurev.immunol.021908.132612 19302047

[B31] Kishimoto T IL-6: from its discovery to clinical applications Int Immunol 2010 22 347 352 10.1093/intimm/dxq030 20410258

[B32] Boone DL Turer EE Lee EG Ahmad RC Wheeler MT Tsui C Hurley P Chien M Chai S Hitotsumatsu O McNally E Pickart C Ma A The ubiquitin-modifying enzyme A20 is required for termination of Toll-like receptor responses Nat Immunol 2004 5 1052 1060 10.1038/ni1110 15334086

[B33] Jacobs MD Harrison SC Structure of an IkappaBalpha/NF-kappaB complex Cell 1998 95 749 758 10.1016/S0092-8674(00)81698-0 9865693

[B34] Wald D Qin J Zhao Z Qian Y Naramura M Tian L Towne J Sims JE Stark GR Li X SIGIRR, a negative regulator of Toll-like receptor-interleukin 1 receptor signaling Nat Immunol 2003 4 920 927 10.1038/ni968 12925853

[B35] Yasukawa H Misawa H Sakamoto H Masuhara M Sasaki A Wakioka T Ohtsuka S Imaizumi T Matsuda T Ihle JN Yoshimura A The JAK-binding protein JAB inhibits Janus tyrosine kinase activity through binding in the activation loop EMBO J 1999 18 1309 1320 10.1093/emboj/18.5.1309 10064597 PMC1171221

[B36] Van Ginderachter JA Movahedi K Hassanzadeh Ghassabeh G, Meerschaut S, Beschin A, Raes G, De Baetselier P: Classical and alternative activation of mononuclear phagocytes: picking the best of both worlds for tumor promotion Immunobiology 2006 211 487 501 10.1016/j.imbio.2006.06.002 16920488

[B37] Raes G Van den Bergh R De Baetselier P Ghassabeh GH Scotton C Locati M Mantovani A Sozzani S Arginase-1 and Ym1 are markers for murine, but not human, alternatively activated myeloid cells J Immunol 2005 174 6561 Author reply 6561–6562 15905489 10.4049/jimmunol.174.11.6561

[B38] Kohler C Allograft inflammatory factor-1/Ionized calcium-binding adapter molecule 1 is specifically expressed by most subpopulations of macrophages and spermatids in testis Cell Tissue Res 2007 330 291 302 10.1007/s00441-007-0474-7 17874251

[B39] Murray PJ Wynn TA Protective and pathogenic functions of macrophage subsets Nat Rev Immunol 2011 11 723 737 10.1038/nri3073 21997792 PMC3422549

[B40] Mege JL Mehraj V Capo C Macrophage polarization and bacterial infections Curr Opin Infect Dis 2011 24 230 234 10.1097/QCO.0b013e328344b73e 21311324

[B41] Hsieh CS Macatonia SE Tripp CS Wolf SF O'Garra A Murphy KM Development of TH1 CD4+ T cells through IL-12 produced by Listeria-induced macrophages Science 1993 260 547 549 10.1126/science.8097338 8097338

[B42] Gately MK Renzetti LM Magram J Stern AS Adorini L Gubler U Presky DH The interleukin-12/interleukin-12-receptor system: role in normal and pathologic immune responses Annu Rev Immunol 1998 16 495 521 10.1146/annurev.immunol.16.1.495 9597139

[B43] Mattner F Ozmen L Podlaski FJ Wilkinson VL Presky DH Gately MK Alber G Treatment with homodimeric interleukin-12 (IL-12) p40 protects mice from IL-12-dependent shock but not from tumor necrosis factor alpha-dependent shock Infect Immun 1997 65 4734 4737 9353058 10.1128/iai.65.11.4734-4737.1997 PMC175679

[B44] MacMicking J Xie QW Nathan C Nitric oxide and macrophage function Annu Rev Immunol 1997 15 323 350 10.1146/annurev.immunol.15.1.323 9143691

[B45] Cai D Deng K Mellado W Lee J Ratan RR Filbin MT Arginase I and polyamines act downstream from cyclic AMP in overcoming inhibition of axonal growth MAG and myelin in vitro Neuron 2002 35 711 719 10.1016/S0896-6273(02)00826-7 12194870

[B46] Wheelock MJ Jensen PJ Regulation of keratinocyte intercellular junction organization and epidermal morphogenesis by E-cadherin J Cell Biol 1992 117 415 425 10.1083/jcb.117.2.415 1373144 PMC2289421

[B47] Suffia I Reckling SK Salay G Belkaid Y A role for CD103 in the retention of CD4+CD25+ Treg and control of Leishmania major infection J Immunol 2005 174 5444 5455 15845457 10.4049/jimmunol.174.9.5444

[B48] Brown GD Taylor PR Reid DM Willment JA Williams DL Martinez-Pomares L Wong SY Gordon S Dectin-1 is a major beta-glucan receptor on macrophages J Exp Med 2002 196 407 412 10.1084/jem.20020470 12163569 PMC2193936

[B49] Teng X Li D Champion HC Johns RA FIZZ1/RELMalpha, a novel hypoxia-induced mitogenic factor in lung with vasoconstrictive and angiogenic properties Circ Res 2003 92 1065 1067 10.1161/01.RES.0000073999.07698.33 12714564

[B50] Martinez FO Helming L Gordon S Alternative activation of macrophages: an immunologic functional perspective Annu Rev Immunol 2009 27 451 483 10.1146/annurev.immunol.021908.132532 19105661

[B51] Swain SL Weinberg AD English M Huston G IL-4 directs the development of Th2-like helper effectors J Immunol 1990 145 3796 3806 2147202

[B52] Moore KW de Waal Malefyt R Coffman RL O’Garra A Interleukin-10 and the interleukin-10 receptor Annu Rev Immunol 2001 19 683 765 10.1146/annurev.immunol.19.1.683 11244051

[B53] Wynn TA IL-13 effector functions Annu Rev Immunol 2003 21 425 456 10.1146/annurev.immunol.21.120601.141142 12615888

[B54] Chieppa M Bianchi G Doni A Del Prete A Sironi M Laskarin G Monti P Piemonti L Biondi A Mantovani A Introna M Allavena P Cross-linking of the mannose receptor on monocyte-derived dendritic cells activates an anti-inflammatory immunosuppressive program J Immunol 2003 171 4552 4560 14568928 10.4049/jimmunol.171.9.4552

[B55] Sparvero LJ Asafu-Adjei D Kang R Tang D Amin N Im J Rutledge R Lin B Amoscato AA Zeh HJ Lotze MT RAGE (Receptor for Advanced Glycation Endproducts), RAGE ligands, and their role in cancer and inflammation J Transl Med 2009 7 17 10.1186/1479-5876-7-17 19292913 PMC2666642

[B56] Daws MR Sullam PM Niemi EC Chen TT Tchao NK Seaman WE Pattern recognition by TREM-2: binding of anionic ligands J Immunol 2003 171 594 599 12847223 10.4049/jimmunol.171.2.594

[B57] Turnbull IR Gilfillan S Cella M Aoshi T Miller M Piccio L Hernandez M Colonna M Cutting edge: TREM-2 attenuates macrophage activation J Immunol 2006 177 3520 3524 16951310 10.4049/jimmunol.177.6.3520

[B58] Owhashi M Arita H Hayai N Identification of a novel eosinophil chemotactic cytokine (ECF-L) as a chitinase family protein J Biol Chem 2000 275 1279 1286 10.1074/jbc.275.2.1279 10625674

[B59] Arora M Chen L Paglia M Gallagher I Allen JE Vyas YM Ray A Ray P Simvastatin promotes Th2-type responses through the induction of the chitinase family member Ym1 in dendritic cells Proc Natl Acad Sci U S A 2006 103 7777 7782 10.1073/pnas.0508492103 16682645 PMC1472521

[B60] Gordon S Alternative activation of macrophages Nat Rev Immunol 2003 3 23 35 10.1038/nri978 12511873

[B61] Piccinini AM Midwood KS DAMPening inflammation by modulating TLR signalling Mediators Inflamm 2010 2010 pii: 672395 10.1155/2010/672395 PMC2913853 20706656

[B62] Li M Carpio DF Zheng Y Bruzzo P Singh V Ouaaz F Medzhitov RM Beg AA An essential role of the NF-kappa B/Toll-like receptor pathway in induction of inflammatory and tissue-repair gene expression by necrotic cells J Immunol 2001 166 7128 7135 11390458 10.4049/jimmunol.166.12.7128

[B63] Van den Bossche J Bogaert P van Hengel J Guerin CJ Berx G Movahedi K Van den Bergh R Pereira-Fernandes A Geuns JM Pircher H Dorny P Grooten J De Baetselier P Van Ginderachter JA Alternatively activated macrophages engage in homotypic and heterotypic interactions through IL-4 and polyamine-induced E-cadherin/catenin complexes Blood 2009 114 4664 4674 10.1182/blood-2009-05-221598 19726720

[B64] Anthony RM Rutitzky LI Urban JF Stadecker MJ Gause WC Protective immune mechanisms in helminth infection Nat Rev Immunol 2007 7 975 987 10.1038/nri2199 18007680 PMC2258092

[B65] Nadeau S Filali M Zhang J Kerr BJ Rivest S Soulet D Iwakura Y de Rivero Vaccari JP Keane RW Lacroix S Functional recovery after peripheral nerve injury is dependent on the pro-inflammatory cytokines IL-1beta and TNF: implications for neuropathic pain J Neurosci 2011 31 12533 12542 10.1523/JNEUROSCI.2840-11.2011 21880915 PMC6703268

[B66] Loke P Gallagher I Nair MG Zang X Brombacher F Mohrs M Allison JP Allen JE Alternative activation is an innate response to injury that requires CD4+ T cells to be sustained during chronic infection J Immunol 2007 179 3926 3936 17785830 10.4049/jimmunol.179.6.3926

[B67] Shechter R London A Varol C Raposo C Cusimano M Yovel G Rolls A Mack M Pluchino S Martino G Jung S Schwartz M Infiltrating blood-derived macrophages are vital cells playing an anti-inflammatory role in recovery from spinal cord injury in mice PLoS Med 2009 6 e1000113 10.1371/journal.pmed.1000113 19636355 PMC2707628

[B68] Barrette B Hebert MA Filali M Lafortune K Vallieres N Gowing G Julien JP Lacroix S Requirement of myeloid cells for axon regeneration J Neurosci 2008 28 9363 9376 10.1523/JNEUROSCI.1447-08.2008 18799670 PMC6671109

[B69] Vargas ME Watanabe J Singh SJ Robinson WH Barres BA Endogenous antibodies promote rapid myelin clearance and effective axon regeneration after nerve injury Proc Natl Acad Sci U S A 2010 107 11993 11998 10.1073/pnas.1001948107 20547838 PMC2900702

[B70] Hikawa N Takenaka T Myelin-stimulated macrophages release neurotrophic factors for adult dorsal root ganglion neurons in culture Cell Mol Neurobiol 1996 16 517 528 10.1007/BF02150231 8879753 PMC11563098

[B71] Kigerl KA Gensel JC Ankeny DP Alexander JK Donnelly DJ Popovich PG Identification of two distinct macrophage subsets with divergent effects causing either neurotoxicity or regeneration in the injured mouse spinal cord J Neurosci 2009 29 13435 13444 10.1523/JNEUROSCI.3257-09.2009 19864556 PMC2788152

[B72] Lu D Goussev A Chen J Pannu P Li Y Mahmood A Chopp M Atorvastatin reduces neurological deficit and increases synaptogenesis, angiogenesis, and neuronal survival in rats subjected to traumatic brain injury J Neurotrauma 2004 21 21 32 10.1089/089771504772695913 14987462

[B73] Pannu R Barbosa E Singh AK Singh I Attenuation of acute inflammatory response by atorvastatin after spinal cord injury in rats J Neurosci Res 2005 79 340 350 10.1002/jnr.20345 15605375

[B74] Angelov DN Waibel S Guntinas-Lichius O Lenzen M Neiss WF Tomov TL Yoles E Kipnis J Schori H Reuter A Ludolph A Schwartz M Therapeutic vaccine for acute and chronic motor neuron diseases: implications for amyotrophic lateral sclerosis Proc Natl Acad Sci U S A 2003 100 4790 4795 10.1073/pnas.0530191100 12668759 PMC153634

[B75] Sicotte M Tsatas O Jeong SY Cai CQ He Z David S Immunization with myelin or recombinant Nogo-66/MAG in alum promotes axon regeneration and sprouting after corticospinal tract lesions in the spinal cord Mol Cell Neurosci 2003 23 251 263 10.1016/S1044-7431(03)00053-8 12812757

[B76] Huang DW McKerracher L Braun PE David S A therapeutic vaccine approach to stimulate axon regeneration in the adult mammalian spinal cord Neuron 1999 24 639 647 10.1016/S0896-6273(00)81118-6 10595515

[B77] Wolf SA Fisher J Bechmann I Steiner B Kwidzinski E Nitsch R Neuroprotection by T-cells depends on their subtype and activation state J Neuroimmunol 2002 133 72 80 10.1016/S0165-5728(02)00367-3 12446010

[B78] Aarli JA Role of cytokines in neurological disorders Curr Med Chem 2003 10 1931 1937 10.2174/0929867033456918 12871095

[B79] Dahle C Ekerfelt C Vrethem M Samuelsson M Ernerudh J T helper type 2 like cytokine responses to peptides from P0 and P2 myelin proteins during the recovery phase of Guillain-Barre syndrome J Neurol Sci 1997 153 54 60 10.1016/S0022-510X(97)00178-0 9455979

[B80] Inoue A Iwahashi T Koh CS Yanagisawa N A study on subpopulation of helper T cells in chronic inflammatory demyelinating polyneuropathy Arerugi 1994 43 1270 1276 7826223

[B81] Deretzi G Pelidou SH Zou LP Quiding C Zhu J Local effects of recombinant rat interleukin-6 on the peripheral nervous system Immunology 1999 97 582 587 10.1046/j.1365-2567.1999.00808.x 10457210 PMC2326882

[B82] Malandrini A Villanova M Dotti MT Federico A Acute inflammatory neuropathy in Charcot-Marie-Tooth disease Neurology 1999 52 859 861 10.1212/WNL.52.4.859 10078742

[B83] Shy ME Arroyo E Sladky J Menichella D Jiang H Xu W Kamholz J Scherer SS Heterozygous P0 knockout mice develop a peripheral neuropathy that resembles chronic inflammatory demyelinating polyneuropathy (CIDP) J Neuropathol Exp Neurol 1997 56 811 821 9210878

[B84] Schmid CD Stienekemeier M Oehen S Bootz F Zielasek J Gold R Toyka KV Schachner M Martini R Immune deficiency in mouse models for inherited peripheral neuropathies leads to improved myelin maintenance J Neurosci 2000 20 729 735 10632602 10.1523/JNEUROSCI.20-02-00729.2000 PMC6772400

[B85] Carenini S Maurer M Werner A Blazyca H Toyka KV Schmid CD Raivich G Martini R The role of macrophages in demyelinating peripheral nervous system of mice heterozygously deficient in p0 J Cell Biol 2001 152 301 308 10.1083/jcb.152.2.301 11266447 PMC2199607

[B86] Qiu J Cai D Dai H McAtee M Hoffman PN Bregman BS Filbin MT Spinal axon regeneration induced by elevation of cyclic AMP Neuron 2002 34 895 903 10.1016/S0896-6273(02)00730-4 12086638

[B87] Kuo HS Tsai MJ Huang MC Chiu CW Tsai CY Lee MJ Huang WC Lin YL Kuo WC Cheng H Acid fibroblast growth factor and peripheral nerve grafts regulate Th2 cytokine expression, macrophage activation, polyamine synthesis, and neurotrophin expression in transected rat spinal cords J Neurosci 2011 31 4137 4147 10.1523/JNEUROSCI.2592-10.2011 21411654 PMC6623531

[B88] Lee YS Hsiao I Lin VW Peripheral nerve grafts and aFGF restore partial hindlimb function in adult paraplegic rats J Neurotrauma 2002 19 1203 1216 10.1089/08977150260338001 12427329

[B89] Kaji R Kiyoshima-Shibata J Nagaoka M Nanno M Shida K Bacterial teichoic acids reverse predominant IL-12 production induced by certain lactobacillus strains into predominant IL-10 production via TLR2-dependent ERK activation in macrophages J Immunol 2010 184 3505 3513 10.4049/jimmunol.0901569 20190136

[B90] Redecke V Hacker H Datta SK Fermin A Pitha PM Broide DH Raz E Cutting edge: activation of Toll-like receptor 2 induces a Th2 immune response and promotes experimental asthma J Immunol 2004 172 2739 2743 14978071 10.4049/jimmunol.172.5.2739

